# Meiotic Chromosome Pairing Is Promoted by Telomere-Led Chromosome Movements Independent of Bouquet Formation

**DOI:** 10.1371/journal.pgen.1002730

**Published:** 2012-05-24

**Authors:** Chih-Ying Lee, Michael N. Conrad, Michael E. Dresser

**Affiliations:** 1 Program in Cell Cycle and Cancer Biology, Oklahoma Medical Research Foundation, Oklahoma City, Oklahoma, United States of America; 2 Department of Cell Biology, University of Oklahoma Health Sciences Center, Oklahoma City, Oklahoma, United States of America; National Cancer Institute, United States of America

## Abstract

Chromosome pairing in meiotic prophase is a prerequisite for the high fidelity of chromosome segregation that haploidizes the genome prior to gamete formation. In the budding yeast *Saccharomyces cerevisiae*, as in most multicellular eukaryotes, homologous pairing at the cytological level reflects the contemporaneous search for homology at the molecular level, where DNA double-strand broken ends find and interact with templates for repair on homologous chromosomes. Synapsis (synaptonemal complex formation) stabilizes pairing and supports DNA repair. The bouquet stage, where telomeres have formed a transient single cluster early in meiotic prophase, and telomere-promoted rapid meiotic prophase chromosome movements (RPMs) are prominent temporal correlates of pairing and synapsis. The bouquet has long been thought to contribute to the kinetics of pairing, but the individual roles of bouquet and RPMs are difficult to assess because of common dependencies. For example, in budding yeast RPMs and bouquet both require the broadly conserved SUN protein Mps3 as well as Ndj1 and Csm4, which link telomeres to the cytoskeleton through the intact nuclear envelope. We find that mutants in these genes provide a graded series of RPM activity: wild-type>*mps3-dCC*>*mps3-dAR*>*ndj1*Δ>*mps3-dNT* = *csm4*Δ. Pairing rates are directly correlated with RPM activity even though only wild-type forms a bouquet, suggesting that RPMs promote homologous pairing directly while the bouquet plays at most a minor role in *Saccharomyces cerevisiae*. A new collision trap assay demonstrates that RPMs generate homologous and heterologous chromosome collisions in or before the earliest stages of prophase, suggesting that RPMs contribute to pairing by stirring the nuclear contents to aid the recombination-mediated homology search.

## Author Summary

Sexual reproduction involves the fusion of gametes, as of a sperm and an egg, to produce the next generation. Each gamete must carry half the number of chromosomes of each parent so that the correct number is restored at fertilization. In order to orient chromosomes properly so that the two chromosomes of each pair (“homologs”) separate to opposite poles in the first meiotic division, the chromosomes first must find one another and align in close proximity (“pairing”) and then be fastened together along their lengths (“synapsis”) in meiotic prophase. Pairing is a poorly understood process that involves movement coupled with a mechanism for recognizing homology. We examine the role played by telomere-promoted chromosome movements (rapid prophase movements, or “RPMs”) and find a correlation between movement and pairing rates, suggesting that RPMs contribute directly to pairing. RPMs cause collisions between nonhomologous as well as between homologous chromosomes, suggesting that RPMs stir the nuclear contents to stimulate recombination-dependent pairing.

## Introduction

Haploidization of the genome for sexual reproduction depends critically on homologous chromosome pairing early in meiotic prophase. How chromosomes pair is a long-standing and largely unanswered question, but pairing requires that chromosomes search for homology and then stabilize homologous interactions to the exclusion of heterologous associations. Given sufficient time, a random walk driven by diffusion, Brownian or metabolic motion might serve to foster homologous chromosome interactions. However, the complexity and efficiency of chromosome pairing has long suggested that the nuclear contents are actively stirred to bring homologous regions into proximity [1]. This notion is supported by the occurrence of well-conserved, rapid meiotic prophase chromosome movements (RPMs; [2]–[7]). These movements are believed to be driven by SUN protein-mediated links through the intact nuclear envelope that connect telomeres to cytoplasmic motors [8], [9]. Defects in SUN genes cause defects both in RPMs and in pairing (reviewed in [10]–[12] and see [13]–[16]).

Telomere-led chromosome positioning contributes to chromosome pairing in a variety of organisms that exhibit different styles of meiotic prophase. Organisms such as mouse, maize and *S. cerevisiae* exhibit a “canonical” meiosis in which synapsis (synaptonemal complex formation [17]) initiates along the paired chromosomes at multiple sites. Concomitant with pairing in these organisms, RPMs drive telomeres to cluster transiently in a limited region of the nuclear envelope, forming the chromosome bouquet [18]–[20]. In *Caenorhabditis elegans*, where synapsis is initiated only at specific telomere-proximal “pairing centers” [21], RPMs similarly are present as the pairing sites accumulate at a common location [13]. In *Schizosaccharomyces pombe*, chromosomes do not synapse but the telomeres are drawn to the spindle pole and remain there throughout meiotic prophase as the whole nucleus is pulled back and forth from one end of the cell to the other by the spindle pole, a process which aligns homologs for recombinational interactions [22]. Thus, despite differing in detail, all these organisms share a stage in which telomeres are brought to a common site.

A long-standing hypothesis is that the bouquet promotes pairing by aligning the chromosomes, but this continues to be a matter of debate [18]–[20]. Observations in several organisms challenge this hypothesis in its simplest form. The bouquet stage follows chromosome pairing in the fungus *Sordaria macrospora* [23] and follows synaptic initiation in female mice [24] and in cattle [25]. However, it remains possible that bouquet formation plays a subtle but important pairing role in these organisms, for example, in testing for and/or promoting pairing of relatively rare laggard chromosomes [20].

In *S. cerevisiae*, the bouquet is absent and pairing is delayed in the mutants *ndj1*Δ [26], *mps3-dNT* (deletion of N-terminal amino acids 2 through 64 of Mps3 [9]) and *csm4*Δ [27]–[30], consistent with a role for the bouquet in pairing. However, RPMs similarly depend on these proteins [27], [29], [31], raising the possibility that RPMs aid pairing directly and, potentially, separately from any RPM role in bouquet formation. By a simple random stirring force model, RPM and pairing rates should be positively correlated, independent of bouquet formation.

Here we examine the role of RPMs in homologous pairing. We extend recognition of RPMs to a period that coincides with pairing early in meiotic prophase and, using a novel chromosome collision trap, show that defects in RPMs decrease collisions between homologous as well as heterologous chromosomes. We find that RPM activities in two additional bouquet-defective *mps3* mutants are intermediate between wild-type and *ndj1*Δ and provide evidence that the RPM reductions result from simple mechanical defects in the linkage between telomeres and cytoplasmic motors. Pairing kinetics in these and other bouquet-defective mutants indicate that pairing correlates with RPMs but not with canonical bouquet formation. We present a “stirring force” model for the role of RPMs in promoting homologous pairing.

## Results

### Partial deletion mutants of *MPS3* provide viable alleles with different meiotic defects

We demonstrated previously that SUN protein Mps3 [32], [33] forms a critical part of the link that connects telomeres with cytoplasmic motors to generate rapid chromosome movements in meiotic prophase in budding yeast [9], [27]. Partial deletion allele *mps3-dNT* removes the intranuclear domain that binds Ndj1 and prevents normal accumulation of Mps3 at the telomeres, presumably largely abrogating a SUN protein-mediated link to the cytoskeleton and eliminating the RPMs. To further test the role of Mps3, we made two additional deletions, of a coiled-coil region composed of residues 240–320 (*mps3-dCC*) which is in the perinuclear lumen and of an acidic region composed of residues 65–145 (*mps3-dAR*) which is intranuclear. Repeated attempts to delete the SUN domain failed to produce viable cells either in our standard laboratory strain (unlike the results in [15]). Deletion *mps3-dCC* is reasonably predicted to eliminate dimerization of Mps3 [34] which we expect to influence RPMs directly. The impact on RPMs of deletion *mps3-dAR* is more difficult to predict as there is growing recognition of the roles of Mps3 and this domain in a wide variety of telomere and DNA double-strand break activities at the nuclear envelope in mitotic cells [35]–[42]. Surprisingly, the meiotic phenotypes of both alleles are relatively mild (see below), but they have provided important insights into the role of RPMs and the meiotic bouquet.

### RPMs are detectable at the onset of meiotic homolog pairing

We assayed for the onset of pairing in strains with homologous loci marked by GFP-tagged spots (concatemers of lacO bound by lacI-GFP fusion protein [43]) where sufficiently close proximity of the two spots, <0.2 µm, causes them to appear as a single spot which is scored as “paired.” A fraction of the population scores as paired prior to meiotic prophase due to somatic pairing in budding yeast [44]. This fraction decreases following induction of meiosis until meiotic pairing causes an increase in the fraction. In our wild-type strains, this increase in pairing starts between t_3_ and t_4_ (where “t_#_” denotes the number of hours following induction of meiosis by transferring cells into sporulation medium; [9], [27] and see below). At t_3_, recombination is in its early stages, as induction of gene conversion has reached only 10–15% of its final levels (see Supplemental Figure 7B in [9]). We looked for synapsis at t_3_ by using immunofluorescence to detect Zip1 protein in spread preparations of nuclei, where short lines of Zip1 mark early synapsis [45]. Among 300 nuclei, none had advanced beyond a spotty Zip1 pattern, indicating that extension of synapsis is largely absent at t_3_.

**Figure 1:**
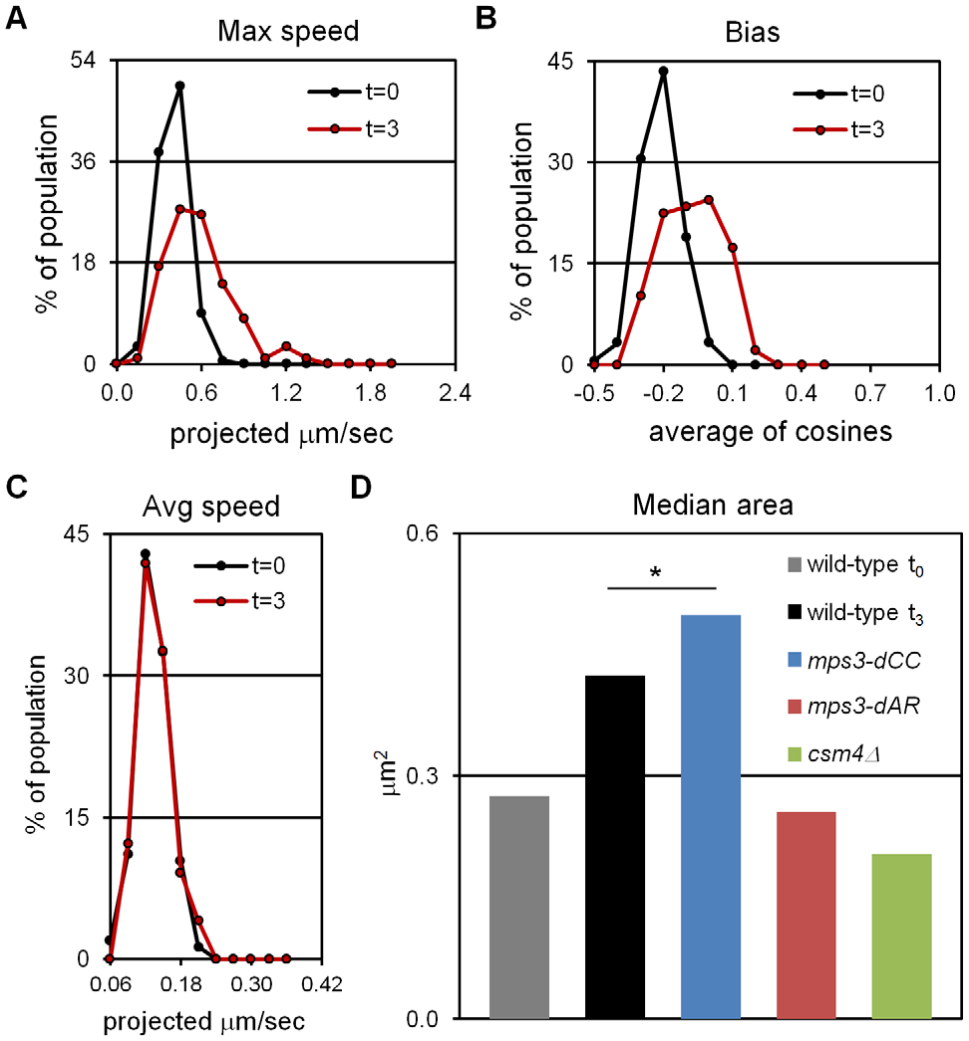
Early RPMs increase the range without increasing the average speed of chromosome movement. Movements were assessed for telomere-adjacent *4*R lacO_256_/lacI-GFP spots in 50 cells at each time-point, in time-lapse datasets acquired at 1 frame per second for 120 seconds total. Only measurements made on cells with two GFP spots, representing unpaired telomeres *4*R, are included. Maximum speed, bias and area are higher at t = 3 (3 hours following the induction of meiosis by transfer into sporulation medium) than at t = 0 (immediately after shift into sporulation medium) but average speed has not yet begun to increase. (A) Histogram of the maximum distances moved in 1 second, for the 119 movements measured for each spot. (B) Histogram of the bias (the average cosine of the angles between each successive movement) for each spot. (C) Histogram of the average speeds for each spot. (D) The median area for the smallest bounding boxes required to enclose all positions of each spot at t_3_. Areas are measured for 60 rather than 120 second intervals to facilitate comparisons with area measures in Figure 6, Figure S1 and [27]. By this measure, RPMs in *mps3-dCC* are not significantly different from wild-type at t_3_ (*) while RPMs are significantly decreased in *mps3-dAR* and *csm4Δ*. Statistical analysis is in the legend to Table 1. Strains used: wild-type (MDY1560XMDY1567), *mps3-dCC* (MDY2580XMDY2759), *mps3-dAR* (MDY2523XMDY2756), *csm4Δ* (MDY2609XMDY2778).

Having established t_3_ as an appropriately early time-point, we assayed for RPMs by acquiring thru-focus time-lapse images, where fluorescence signal is in essence projected onto a 2D plane orthogonal to the Z, or focusing, axis [46]. Previous results suggested that rapid movements were infrequent at t_3_ (see Figure 2 in [27]), so we acquired images every 1 second for 120 seconds total, rather than our typical 60 seconds total, in a wild-type strain homozygous for a GFP spot adjacent to telomere *4*R. We chose this locus because chromosome *4* and its right arm are relatively long, presumably buffering the movements of *4*R telomeres from potential centromere effects and from opposing pulling by the *4*L telomeres [27]. Movements were quantified in the time-lapse images using parameters that reflect the speed of the movements (maximum and average speed) and the tendency of the spots to move away from their starting positions (bias and area). All measurements are made on spots projected along the Z (focusing) axis onto a plane, as required by thru-focus image acquisition [27]. Maximum speed indicates the single longest step taken by a spot during the time-lapse acquisition, and average speed indicates the mean for all steps, in units of (projected) microns per second. Bias, a unitless measure adapted from studies of bacterial motility [47], is calculated as the average of the cosines of the angles made by the pairs of vectors representing successive movements (bias is 0 for random movement, <0 for the tendency to remain in place and >0 for tendency to move away from the starting position). Area is the area of the minimum bounding box required to enclose all (projected) spot positions, in units of square microns, and represents the combined effects of average speed and bias. Except for average speed, all measures reveal significant increases in telomere mobility from t_0_ to t_3_, indicating the presence of RPMs at t_3_ (Figure 1A, 1B, 1D and Table 1; median values are compared for measurements of area, see [27]). The average speed per nucleus shows no increase from t_0_ to t_3_ (Figure 1C, Kolmogorov-Smirnov test P = 0.623), indicating that while these early movements broaden the range of travel of the telomeres, and occasionally are punctuated by faster movements, there is no net increase in average speed as compared with movements prior to induction of meiosis.

**Table 1:** Quantified mutant phenotype values.

*Measure* a	wild-type	*mps3-dCC*	*mps3-dAR*	*ndj1*Δ	*csm4*Δ	*mps3-dNT*
RPM area t_3_, unprd^b^	0.42	0.50	0.26	NA	0.20	NA
Pairing rate^c^	12.1	10.8	9.2	5.5	NA	NA
RPM area t_4_, prd^d^	2.12	1.40	0.76	0.34	0.12	0.12
RPM area t_4_, unprd^e^	1.22	0.64	0.28	0.25	0.10	0.18
Viable spore prod.^f^	2.8	1.6	1.4	1.4	0.5	0.9
Disomy for chr*3* g	3.0×10^−4^	4.9×10^−4^	5.9×10^−3^	1.7×10^−2^	1.9×10^−2^	NA
PSCS for chr*7* h	4.50	3.33	9.17	9.13	9.67	12.33
“Bouquet” in *rec8*Δ^i^	16.6	6.1	5.4	4.9	NA	NA
TEL-SPB in *rec8*Δ^j^	18.4	9.1	9.2	6.6	NA	NA
TEL cluster in *rec8*Δ^k^	83.5	63.4	35.4	45.2	NA	NA

a “RPM area” is the area in square microns of the smallest bounding box required to enclose all positions of a spot in 60 successive time-lapse images made at 1 acquisition per second.

b Median RPM area values for unpaired spots in units of µm^2^/minute. RPM area for wild-type at t_0_ is 0.28; each pairwise comparison with wild-type at t_3_ is significantly different at P<0.001 by Kolmogorov-Smirnov test, except for *mps3-dCC* where P = 0.64.

c Median values of change in percent paired from t_3_ to t_5_, in units of percent paired per hour. By Mann-Whitney U test, the P values for the differences between wild-type and mutants are 0.066, 0.034 and 0.0025, respectively, and for *mps3-dAR versus ndj1*Δ is 0.025.

d Median RPM area values for paired spots in units of µm^2^/minute. By Mann-Whitney U test, the P values for the differences between wild-type and the mutants are 0.22, 0.052, 3.6×10^−5^, 7.9×10^−7^, and 9.3×10^−5^, respectively.

e Median RPM area values for unpaired spots in units of µm^2^/minute. By Mann-Whitney U test, the P values for the differences between wild-type and the mutants are 1.2×10^−5^, 3.7×10^−16^, 5.8×10^−17^, 2.1×10^−31^, and 1.4×10^−26^, respectively.

f Viable spore production is estimated by multiplying the mean fractions of asci with 3 or 4 spores formed per sporulating cell by the mean number of viable spores in 4-spored asci, which provides a rough estimate of the number of viable spores produced per sporulating cell. By *chi*-*square*, wild-type sporulation (number of asci formed per sporulating cell) and spore viability (Figure 7A, 7B) each differs from all mutants by P<10^−4^.

g Mean values of disomic colonies per total number of viable colonies. By *t*-test, wild-type is not significantly different from *mps3-dCC* (P = 0.21) but differs from *mps3-dAR* with P = 1.0×10^−4^ and P values are smaller for the other mutants.

h Mean values of cells with premature sister chromatid separation of chromosome *7*, as percent of population. By *t*-test, wild-type is not significantly different from *mps3-dCC* (P = 0.26) but differs from *mps3-dAR* with P = 0.016 and from the other mutants with P<0.01. For *mps3-dNT versus csm4*Δ, P = 0.075.

i Fraction of population with telomere cluster and spindle-pole proximity values of less than 0.6, roughly corresponding to the cut-offs between bouquet and non-bouquet nuclei, when in the *rec8*Δ background. By *chi-square*, *rec8*Δ alone (in the “wild-type” column of the table) differs from each of the 3 double mutants with P<0.005; the 3 double mutants are not statistically significantly different from one another.

j Fraction of population with telomere to spindle-pole proximity values of less than 0.6, roughly corresponding to the cut-off between bouquet and non-bouquet nuclei, when in *rec8*Δ background. By *chi-square*, *rec8*Δ alone (in the “wild-type” column of the table) differs from each of the 3 double mutants with P<0.02; the 3 double mutants are not statistically significantly different from one another.

k Fraction of population with telomere cluster values of less than 0.6, roughly corresponding to the cut-off between bouquet and non-bouquet nuclei, when in *rec8*Δ background. By *chi-square*, *rec8*Δ alone (in the “wild-type” column of the table) differs from each of the 3 double mutants with P<10^−7^. The *rec8Δ mps3-CC* mutant shows more clustering than *rec8Δ ndj1*Δ (P = 0.0003); the *rec8Δ mps3-dAR* mutant shows less clustering than *rec8Δ ndj1*Δ (P = 0.05).

NA – Not available.

Because RPMs are associated with increased average speeds at later time-points [27], we asked whether the early movements represent *bona fide* RPMs by deletion of the known RPM gene *CSM4* [27]–[30] and found that the early movements clearly are impaired (Figure 1D, Table 1). Thus, a *CSM4*-dependent increase in the range of telomere movement appears prior to the increase in average speed that has been reported to occur in leptotene cells [48]. Clearly, RPMs accompany the early stages of meiotic chromosome pairing.

### Early prophase RPMs promote chromosome collisions

**Figure 2:**
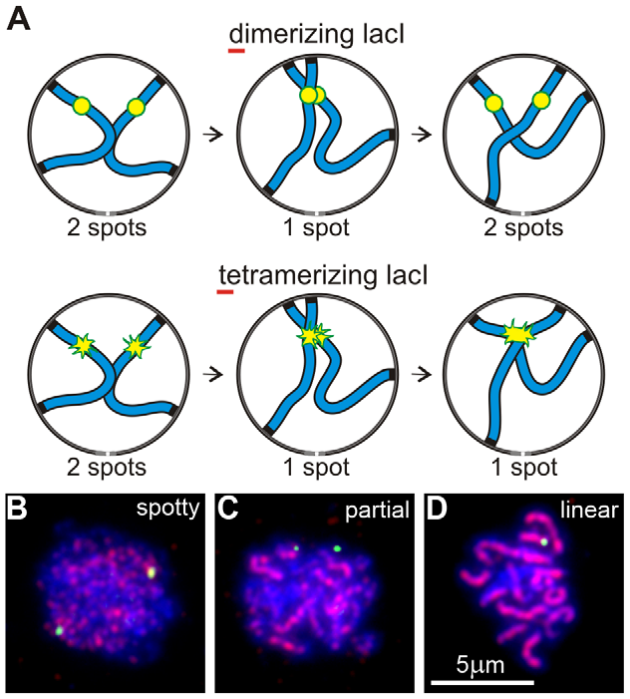
An assay for trapping chromosome collisions. (A) Diagram of an assay designed to trap chromosome collisions. A pair of homologs is represented as blue lines. lacO_256_ concatemers are decorated with dimerizing lacI-GFP in a control strain and with tetramerizing lacI-GFP in the experimental strain (represented as yellow/green spots in the diagrams). Collision between sites with the concatemers leads to stable formation of a single spot with tetramerizing lacI-GFP; dimerizing lacI-GFP allows subsequent separation of the sites so that two spots are again visible. (B–D) Examples of nuclei (DNA labeled with DAPI, blue) at different stages of meiotic prophase as determined by the pattern of signal from immunolocalized Zip1 (red). The yellow-green spots are lacI-GFP-decorated lacO concatemers at the middle of chromosome arms *5*L. Shown are examples of the majority of nuclei at the different stages, i. e., where pairing has not yet occurred in B and C but is evident in D. Nuclei scored as positive in the collision trap assay have Zip1 signal distribution as in B, *i. e.*, have spots but no distinct lines of Zip1, but in addition have only a single GFP spot, as in D.

We developed a “collision trap” assay to ask whether RPMs could promote interactions between specific pairs of chromosome loci. In the absence of stabilization by recombination intermediates or synapsis, interactions between interstitial chromosome loci (non-centromeric, non-telomeric) are expected to be transient. To stabilize these interactions and enable their detection, we took advantage of the ability of tetramerizing lacI protein to link lacO DNA concatemers on separate chromatids [49]. We inserted lacO concatemers in the middle of the left arms of chromosomes *5* and *7*, in strains expressing either dimerizing-lacI-GFP, which does not connect lacO arrays on different chromosomes, or tetramerizing-lacI-GFP which establishes stable, strong interactions with other tetramerizing-lacI-GFP (the principle of this assay is diagrammed in Figure 2A). Prior to the induction of sporulation, the strains were stored and grown in IPTG (Isopropyl-βD-1-thiogalactopyranoside) to block lacI binding to lacO sites. This prevented trapping chromosome-chromosome interactions during mitotic growth which destabilizes the marked chromosomes (data not shown). Our collision trap is similar in principle to one based on Cre/loxP recombination in which transient collisions during meiotic prophase give rise to recombination products that are detected in viable spores [50], but adds the capability to detect interactions in relation to specific stages in prophase. The pattern of Zip1 label was used to identify nuclei that were in early meiotic prophase (Zip1 spots present) but had not yet begun to extend synapsis (Zip1 lines absent; Figure 2B–2D).

**Figure 3:**
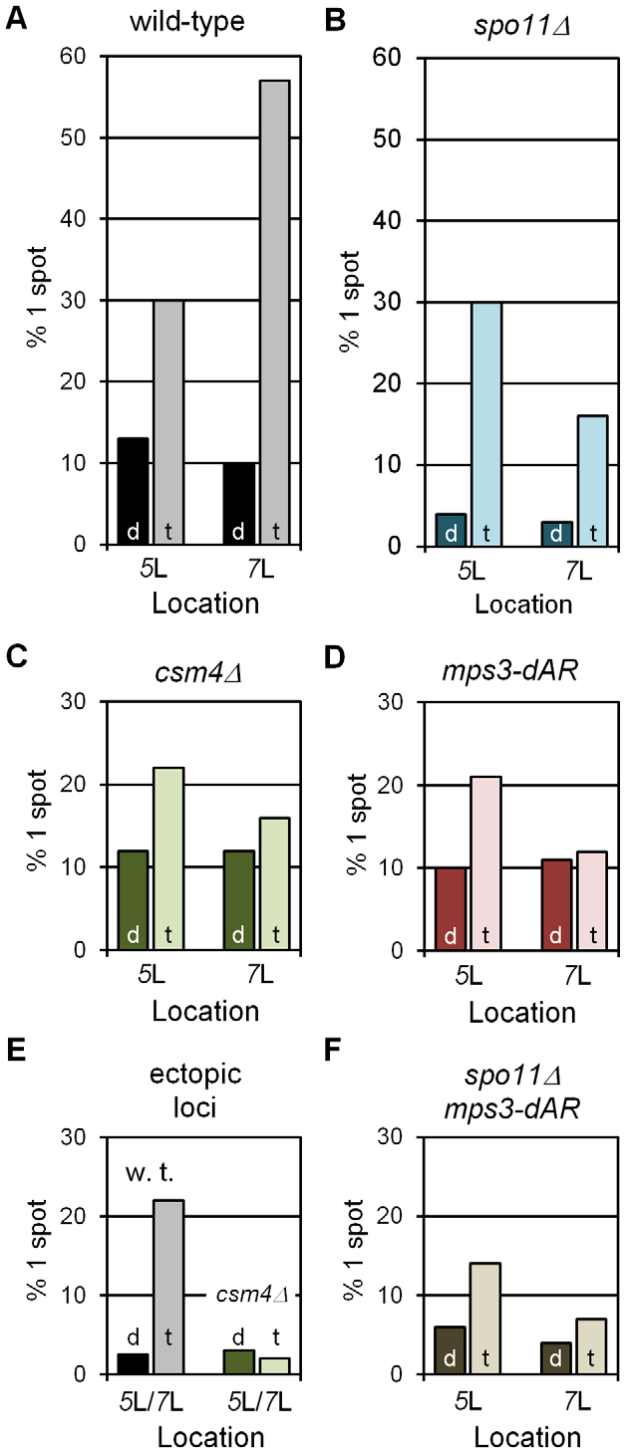
RPMs cause chromosome collisions coincident with pairing. All collision trap assays were performed with pairs of lacO concatemers at interstitial homotopic loci (*5*L or *7*L) or ectopic loci (*5*L/*7*L) and with dimerizing (“d”) or tetramerizing (“t”) lacI-GFP. At least 200 nuclei were scored for each data point for the presence of 1 GFP spot, indicating a pairing or collision event, or 2 separate GFP spots, indicating a separation of ∼0.2 µm or more (see Figure 2C–2E). In wild-type and in *spo11*Δ, where RPMs are robust, tetramerizing lacI-GFP traps collisions that are lost in dimerizing lacI-GFP (A, B). RPM mutants *csm4*Δ and *mps3-dAR* reduce the numbers of collisions whether homotopic (C, D) or ectopic (F), and in the absence of meiotic recombination (E). Statistical analyses are described in the text. Strains used are listed in Table S2. (A–D, F) Results for homotopic collisions in wild-type and mutant backgrounds, as labeled. (E) Results for ectopic collisions in wild-type (*w. t.*) *vs. csm4*Δ. The levels of single spots in dimerizing lacI-GFP in E set an upper limit on the background from chance colocalization of homotopic spots, and in F for heterotopic spots.

Early meiotic prophase nuclei showing one rather than two GFP spots can arise from vegetative pairing, dimerizing-lacI-mediated association, chance overlap, undetected synapsis, loss of one of the lacO concatemers or stabilization of association by flanking early recombinational interactions. Nevertheless, in wild-type control cells, the fractions of single-spot nuclei in dimerizing-lacI-GFP was relatively low, 13% and 10% for chromosome *5* and chromosome *7*, respectively (black bars in Figure 3A). The fractions of single-spot nuclei were considerably higher in tetramerizing-lacI-GFP, 30% and 57% for chromosome *5* and chromosome *7*, respectively (gray bars in Figure 3A; chi square P values of 4.3×10^−7^ and 2.6×10^−55^, respectively). Control genetic experiments ruled out higher levels of concatemer loss in the strains evaluated for tetramerizing lacI effects, and also excluded increased flanking recombination in tetramerizing *versus* dimerizing lacI-GFP in meiosis (data not shown), leaving us to conclude that tetramerizing-lacI-GFP stabilizes collisions that otherwise would be unstable prior to synapsis.

We next asked whether the levels of single-spot nuclei depended on Spo11, which initiates recombination by generating DNA double-strand breaks, a requirement for the development of chromosome axial elements and synapsis [51], [52]. The fractions of single-spot nuclei in the control dimerizing-lacI-GFP experiments for both loci are lower in *spo11*Δ than in wild-type (dark blue bars in Figure 3B), and are lower in tetramerizing-lacI-GFP for chromosome *7* but not for chromosome *5* (light blue bars in Figure 3B). These results suggest that recombination, axial element development (which may contribute to chromosome stiffness) and/or synapsis, which occur in wild-type but not in *spo11*Δ, play a role in generating stable interactions in early prophase; furthermore, one or more of these processes apparently affects mid-arm *5*L and *7*L differentially (possibly because of their different lengths). Nevertheless, in *spo11*Δ single-spot fractions are higher in tetramerizing-lacI-GFP than in dimerizing-lacI-GFP for chromosomes *5* and *7* (chi square P values of 3.5×10^−40^ and 2.5×10^−14^, respectively), indicating trapped collisions. Additionally, in the presence of tetramerizing-lacI-GFP, but not of dimerizing-lacI-GFP, the fractions of single-spot nuclei in *spo11*Δ increase from earlier to later stages (determined by increased Zip1 signal and presence of polycomplexes in later nuclei), as would be expected for ongoing collision trapping even in the absence of recombination and synapsis (the same is seen for *spo11Δ mps3-dAR*; data not shown).

With controls in place, we asked whether defective RPMs lowered the fractions of single-spot nuclei for homologous loci in tetramerizing-lacI-GFP. As expected, decreases in single-spot nuclei were seen for *csm4*Δ (Figure 3C; for *5* and *7*, chi square P values of 8.0×10^−2^ and 1.2×10^−16^, respectively) and for *mps3-dAR* (Figure 3D; for *5* and *7*, chi square P values of 5.0×10^−2^ and 9.9×10^−16^, respectively). Similarly, in the *spo11Δ mps3-dAR* double mutant (*spo11Δ csm4*Δ not tested), the single-spot fractions are lower than for either single mutant (compare Figure 3E with Figure 3B and 3D; for *5* and *7*, comparing *spo11*Δ with *spo11Δ mps3-dAR*, chi square P values are 4.8×10^−4^ and 1.4×10^−2^, respectively). It was possible that the weaker RPMs in *mps3-dAR* might allow tetramerizing lacI-stabilized collisions to accumulate to higher levels (if RPMs disrupted these associations) but the opposite was seen, consistent with *mps3-dAR* reducing the numbers of collisions. This result is consistent with the decreased homologous interaction measured for *spo11Δ ndj1*Δ as compared with either single mutant [53]. In each RPM mutant, levels for chromosome *7* are reduced below those for chromosome *5*, as for *spo11*Δ alone. Finally, we tested the impact of RPMs on single-spot nuclei for heterologous loci, with lacO concatemers on one chromosome *5* and one chromosome *7*, by comparing the single-spot fractions in wild-type *versus csm4*Δ (*mps3-dAR* was not tested). As expected, background control levels are lower than for homologous loci (compare black bars in Figure 3F and Figure 3A; chi square for *5/7* versus *7/7* P = 1.2×10^−2^) and fewer single-spot nuclei are found in *csm4*Δ than in wild-type (light bars in Figure 3F; chi square P = 1.4×10^−6^).

These interaction measures are limited to early prophase nuclei where synapsis, specifically the extension of Zip1 spots into lines, has not begun and do not measure the interactions that presumably occur later during the extended prophase characteristic of RPM mutants. Thus, the observation of reduced heterologous interactions reported here do not contradict observations of increased ectopic recombination in *ndj1*Δ [54], [55] and are consistent with the relatively late appearance of ectopic recombination products in *ndj1*Δ and *csm4*Δ [28], [29]. These results are consistent with RPMs fostering homologous and heterologous chromosome interactions by moving chromosomes through the nucleus.

### RPM activity correlates with the kinetics of homolog pairing

**Figure 4:**
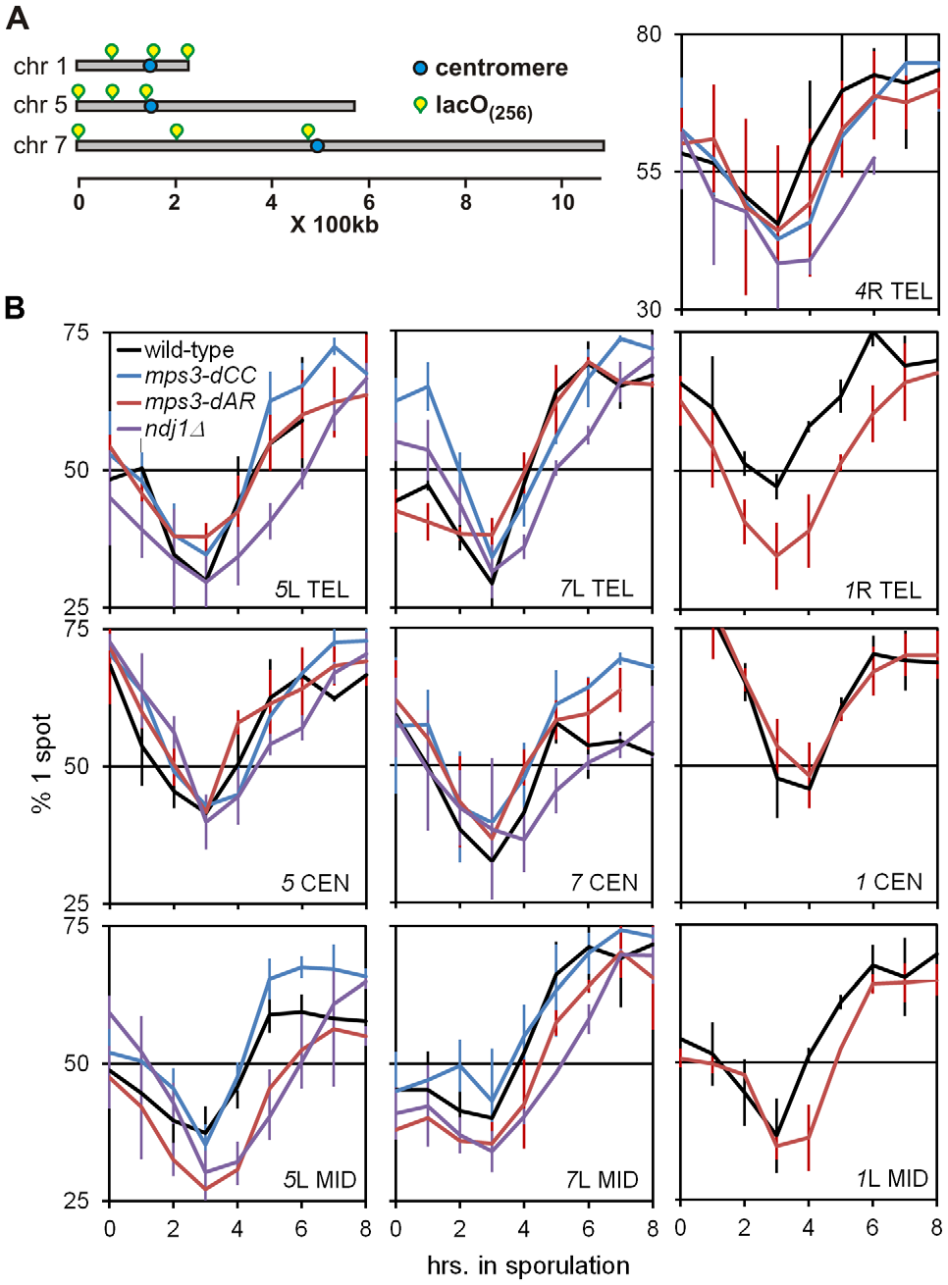
Delays of pairing/synapsis in RPM mutants vary among chromosome loci. (A) Diagram of lacO concatemer positions on chromosomes *1*, *5* and *7* scored for pairing frequency. The *4*R concatemer (not shown) is adjacent to the telomere. (B) Comparison of kinetics of close pairing at different sites on chromosomes *1*, *4*, *5* and *7*. Error bars are 1 standard deviation. Pairing is most delayed in *ndj1*Δ with *mps3-dCC* and *mps3-dAR* appearing to have no or intermediate delays, depending on the site. With only two exceptions, *mps3-dAR* at *5*L telomere and *ndj1*Δ at *7* centromere, all sites are minimally paired at t_3_. Statistical analysis of summary estimates of pairing rates (see text) is in the legend to Table 1. Strains used are listed in Table S3.

We next quantified chromosome pairing rates and RPM activities and tested whether these are correlated. We examined pairing kinetics for a variety of homotopic sites (diagrammed in Figure 4A, plus a *4*R telomere-adjacent site, not illustrated) tagged with (dimerizing) lacI-GFP on lacO concatemers, scoring for one spot (paired) or two spots (unpaired). For each combination of genetic background and homotopic site, samples of 200 living cells were scored at hourly intervals following the shift into sporulation medium, in 3 or more independent experiments (Figure 4B).

The quantitative analysis of pairing kinetics is complicated by two major factors. First, progression through meiosis is not perfectly synchronous because asynchronous mitotic cells are shifted to sporulation medium. Second, some fraction of the single-spot cells represent chance overlap of homologous regions, mitotically paired homologous regions [44], clustered centromeres [56], [57] and/or clustered telomeres [57]–[59], giving rise to large fractions of single-spot cells prior to meiosis and obscuring the lowest levels of meiotic pairing *per se*. These factors contribute to the variation indicated by the error bars (Figure 4B). Another potential complicating factor is that the mutants could delay entry into meiotic prophase. However, since no such delay has been observed with *ndj1*Δ, *mps3-dNT* or *csm4*Δ [9], [27] and pairing increases begin from t_3_ for all genotypes and loci, this possibility does not seem significant. In order to compare pairing rates of different genotypes and at different loci, rates were estimated simply by subtracting the t_3_ from the t_5_ fraction and dividing by 2 to give the rates in percent paired per hour (Table 1).

**Figure 5:**
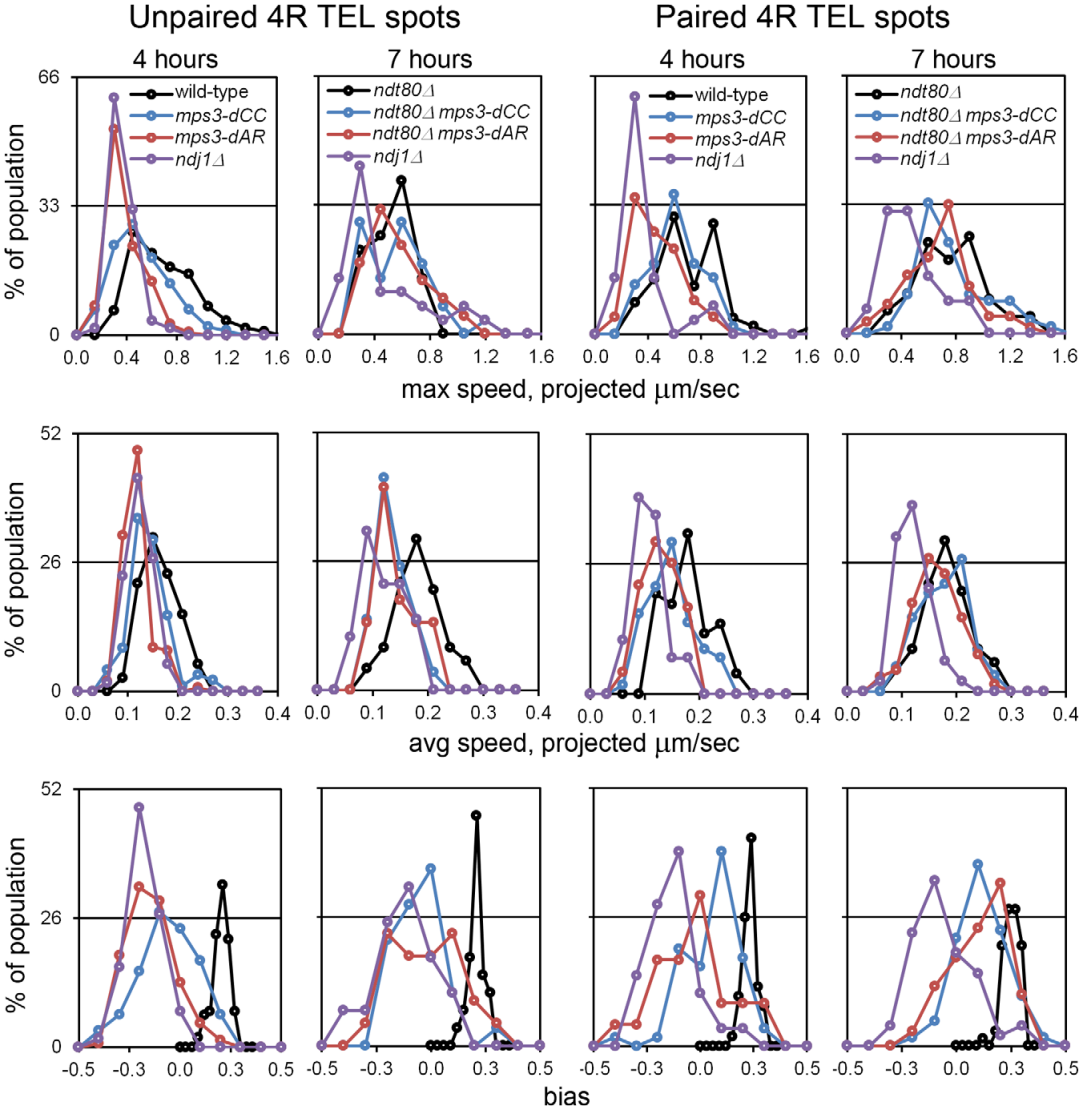
RPMs in the *mps3-dAR and mps3-dCC* mutants are intermediate between *ndj1*Δ and wild-type early in prophase and approach wild-type levels following pairing when prophase exit is blocked. Histograms are shown to display the ranges and changes in RPMs with time by measures of maximum speed, average speed and bias. All measurements are for GFP-tagged spots adjacent to the *4*R telomere and are accumulated separately for nuclei where there are 2 spots (unpaired, left columns) or 1 spot (paired, right columns). All populations are in early to middle meiotic prophase at 4 hours. To compare with the later 7 hour time-point of *ndj1*Δ, when many cells in the other strains would already be undergoing the meiotic divisions, *ndt80*Δ is added to the wild-type, *mps3-dCC* and *msp3-dAR* backgrounds to hold the strains in meiotic prophase and allow the development of the fastest possible RPMs. Note that the “bias” measure, the average of the cosines of the angles made between successive movements, is unitless. Statistical analyses of median values from t_4_ are in the legend to Table 1. Strains used: wild-type (MDY1560XMDY1567), *mps3-dCC* (MDY2580XMDY2759), *mps3-dAR* (MDY2523XMDY2756), *ndj1*Δ (MDY2294XMDY1560), *ndt80*Δ (MDY2984XMDY3021), *ndt80Δ mps3-dCC* (MDY3020XMDY3022), *ndt80Δ mps3-dAR* (MDY3047XMDY3049).

RPM activity was measured in time-lapse movies at t_4_ in cells with Tub1-GFP marking the spindle pole body and GFP-spot markers adjacent to chromosome *4*R telomere (Video S1). Telomere movements are fastest in wild-type, slightly slower in *mps3-dCC*, slower still in *mps3-dAR* and slowest in *ndj1*Δ. This is most apparent visually when comparing nuclei that have a single telomere spot. The differences are seen more clearly by quantifying the movements at t_4_ and, in order to allow the RPMs to develop fully, at t_7_ (*ndt80*Δ was introduced to prevent wild-type, *mps3-dCC* and *mps3-dAR* strains from exiting prophase before 7 hours; Figure 5). These data are inherently complex but, when the peaks and skewness of the histogram curves are compared, there is an evident trend to RPM activity where wild-type>*mps3-dCC*>*mps3-dAR*>*ndj1*Δ.

**Figure 6:**
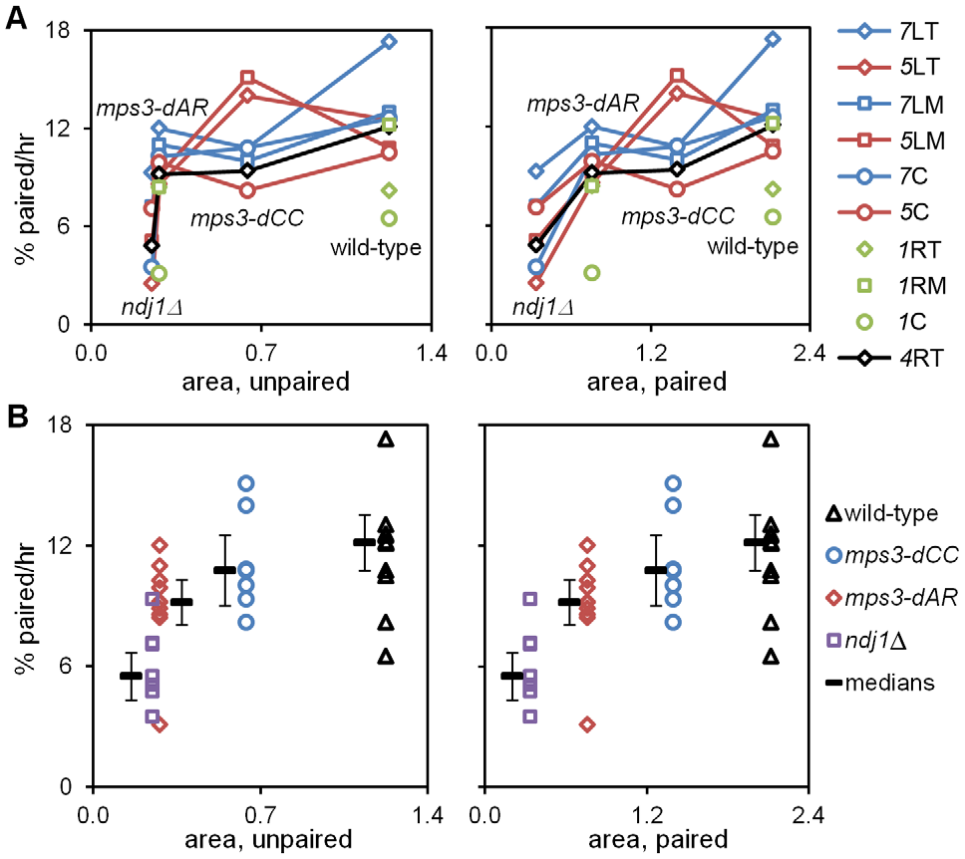
Pairing rates are positively correlated with RPMs. Pairing rates (Figure 4, Table 1; change in the percent of the population with 1 (paired) spot rather than 2 (unpaired) spots) and RPM area measures (Table 1; square microns of bounding box that encloses all spot positions in 60 consecutive time-lapse frames) for telomere *4*R are graphed to display the behavior of individual loci (A) and of individual genotypes (B). Paired telomeres tend to move faster and further than unpaired telomeres in wild-type, *mps3-dCC* and *mps3-dAR* (but the reverse is true for average and maximum speeds in *ndj1Δ*; see [27], Figure 5 and Figure S1). Error bars in B are average absolute deviation from the median. Strains used are the same as for Figure 4 and are listed in Table S3. (A) Pairing rates for each locus (key at right) graphed against RPM area for unpaired (left) and paired (right) telomeres *4*R. Chromosome *1* pairing was determined only for wild-type and *mps3-dAR*. Lines connect values for the same locus in different genetic backgrounds. (B) Median pairing rates for each genotype (thick black lines) are shown adjacent to the individual values, graphed against RPM area for unpaired (left) and paired (right) telomeres *4*R.

We compared RPM activities at t_4_ to the rate of pairing between t_3_ and t_5_. Since the RPM values in many of the datasets are not normally distributed, the median values of the RPMs (which generally are very close to the mean values, data not shown) are used in comparing RPMs to pairing rates. The median area measures for paired and unpaired *4*R telomeres for the mutants are all significantly lower than wild-type with the exception of the paired telomere area for *mps3-dCC* (Table 1). RPM areas are graphed against pairing rates for each locus/genotype combination, separated into those nuclei where the chromosome *4*R telomere spots used to measure the area are unpaired *versus* paired (Figure 6). These data are plotted to emphasize either the behavior of individual loci (Figure 6A) or the variation in the pairing rates by genotype (Figure 6B). Paired (synapsed) telomeres generally have more robust RPMs than unpaired telomeres in wild-type cells but the reverse has been seen for RPMs in bouquet gene mutants, including *mps3-dNT* [27]. The partially defective *mps3-dCC* and *mps3-dAR* are like wild-type rather than *mps3-dNT* in having more robust RPMs when paired. Plots of the medians for each of the other RPM parameters also show a positive correlation with pairing rates (Figure S1).

### Defects in RPM activity correlate with defects in meiotic outcome

**Figure 7:**
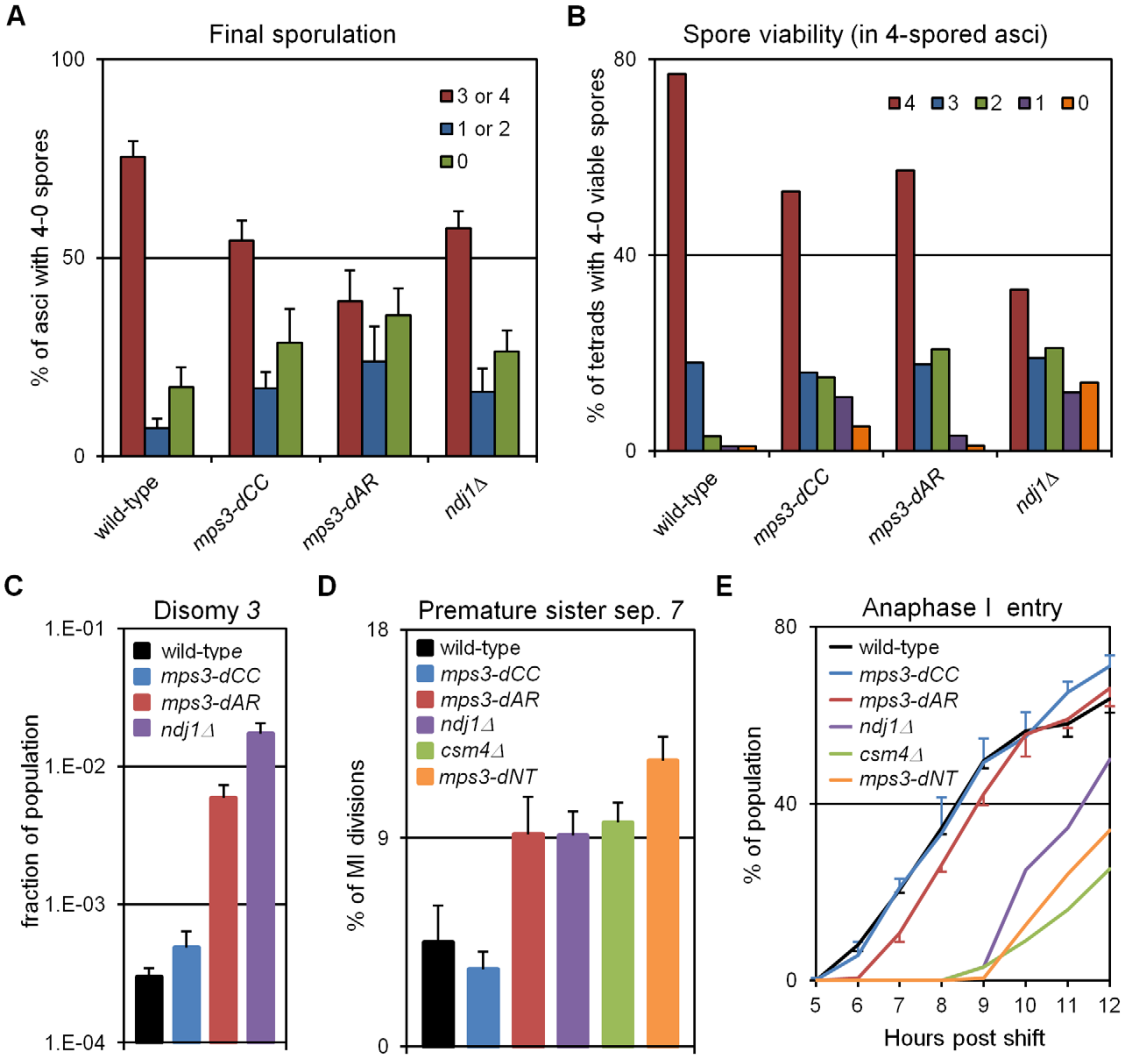
Defects in production of viable spores, proper chromosome segregation, and time to enter the first meiotic division increase with defects in RPMs. (A) Percent of cells with 3-4, 2-1 or 0 spores (the latter mainly representing cells that failed to enter or complete sporulation) after 2 days in sporulation medium. Final sporulation levels for wild-type (MCY506XMCY507), *mps3-dCC* (MCY1378XMCY1379), *mps3-dAR* (MCY1512XMCY1513) and *ndj1*Δ (MCY422XMCY423) are 83%, 71%, 63% and 74%, respectively. Statistical analysis is in the legend to Table 1. (B) Percent of 4-spored asci with 4, 3, 2, 1 or 0 viable spores. Total spore viabilities for wild-type, *mps3-dCC*, *mps3-dAR* and *ndj1*Δ are 92%, 75%, 87% and 62%, respectively. Statistical analysis is in the legend to Table 1. (C) Meiotic missegregation measured by the presence of an extra chromosome *3* in viable spores. Strains used: wild-type (MDY493XMDY494), *mps3-dCC* (MCY1370XMCY1380), *mps3-dAR* (MCY1584XMCY1588) and *ndj1*Δ (MCY420XMCY421). (D) Premature separation of sister chromatids of chromosome *7* in the first meiotic division assayed cytologically using a GFP-tagged spot adjacent to the centromere. Strains used: wild-type (MDY2828XMDY2798), *mps3-dCC* (MDY2843XMDY2825), *mps3-dAR* (MDY2846XMDY2823), *ndj1*Δ (MDY2826XMDY2820), csm4Δ (MDY3042XMCY1539), mps3-dNT (MDY2834XMDY2821). (E) Entry into the first meiotic division was assayed by DAPI staining of DNA in fixed whole cells as the appearance of stretching apart of the dividing DNA mass during the first meiotic division or the presence of two or more nuclear DNA masses in a single cell. Strains used: wild-type (MCY506XMCY507), *mps3-dCC* (MCY1378XMCY1379), *mps3-dAR* (MCY1512XMCY1513), *ndj1*Δ (MCY422XMCY423), *csm4*Δ (MCY1536XMCY1539), *mps3-dNT* (MCY1401XMCY1407). All error bars represent 1 standard deviation.

We expected that events that followed meiotic prophase would be similarly correlated with RPMs, and this is largely true. Each of the mutants makes fewer 4-spored asci with 4 viable spores than wild-type, and in *mps3-dAR* final sporulation is slightly lower but spore viability is slightly higher than in *mps3-dCC* or *ndj1*Δ (Figure 7A–7B; measures combined in Table 1 to estimate viable spore production). Viable spore production in the different genotypes, approximated by multiplying the fraction of cells that make asci with 3 or 4 spores times the mean spore viability in 4-spored asci, correlates directly with RPM activities (Table 1; values for *csm4*Δ and *mps3-dNT* are from published data [9], [27]). Similarly, missegregation of chromosome *3* to give disomes in viable spores (assay described in [60]) correlates directly with increases in RPM defects (Figure 7C; Table 1).

Genetic assays, requiring viable spores, have shown elevated premature sister chromatid separation (PSCS) in *ndj1*Δ [60], [61] but not in *csm4*Δ [29]). In order to avoid the requirement for viable spores, we assayed PSCS cytologically in strains with one chromosome *7* marked with a centromere-adjacent lacO/lacI-GFP spot. Anaphase I cells were identified by DAPI staining and PSCS was scored if the sister GFP spots were clearly separated (Figure 7D, Table 1). PSCS is not elevated in *mps3-dCC*, is elevated equally over wild-type levels in *mps3-dAR*, *ndj1*Δ and *csm4*Δ, and is slightly higher still in *mps3-dNT*. By this measure, PSCS levels are only roughly correlated with RPMs and may be elevated in the mutants for reasons not directly related to RPMs, potentially because of defects in sister chromatid cohesion which has been reported for *ndj1*Δ and *mps3-dNT* [9]. The wild-type level of apparent PSCS (4.5%) is higher than expected given >90% spore viability in 4-spored asci and may reflect crossing over between the centromere and the lacO/lacI-GFP marker.

Progression past meiotic prophase was measured in DAPI stained cells by scoring for separation of the nucleus into two or more masses (Figure 7E). Wild-type and *mps3-dCC* are indistinguishable but *mps3-dAR* shows a ∼1 hour delay and the more defective RPM mutants are still further delayed. With the exception of *mps3-dCC*, where the defects in sporulation and spore viability seem disproportionately strong with respect to the small increase in chromosome missegregation and no prophase delay, the mutant phenotypes outlined above generally trend with RPM defects.

### Short chromosomes are the last to synapse in wild-type and in RPM mutants

**Figure 8:**
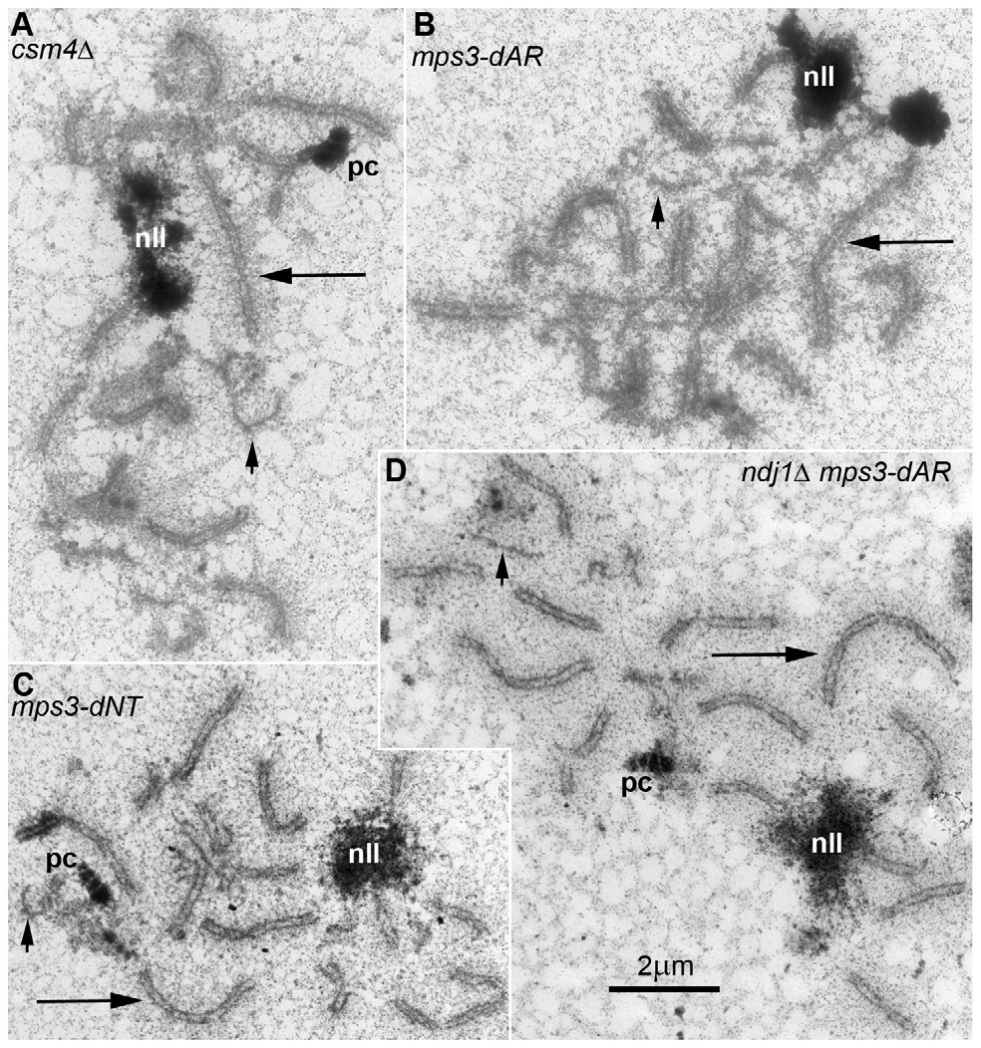
In all RPM mutants, short chromosomes frequently remain unsynapsed when long chromosomes have finished synapsis. Electron micrographs of silver-stained, spread, flattened meiotic prophase nuclei from (A) *mps3-dAR* (MCY1512XMCY1513) at t_6_, (B) *mps3-dNT* (MCY1401XMCY1407) at t_8_, (C) *ndj1Δ mps3-dAR* (MCY1510XMCY1511) at t_8_ and (D) *csm4*Δ (MCY1536XMCY1539) at t_8_. Electron-dense lines are the silver-stained chromosome axes which, when aligned in pairs at uniform spacing, mark completed synapsis to form synaptonemal complexes. Long arrows indicate synapsed long chromosomes and short arrows indicate unsynapsed short chromosome axes. Nucleoli are indicated by “nll.” Polycomplexes, which are commonly found in nuclei that are delayed in synapsis, are indicated by “pc.”

In *ndj1*Δ, completion of synapsis is delayed, with relatively short chromosomes being the last to synapse ([26], [60]. A similar observation has been made for recombination mutants *dmc1*Δ and *rad51*Δ [62]). We prepared silver-stained spreads of mutant meiotic nuclei at relatively late time-points that coincide with entry into the first meiotic division and examined 50 or more nuclei from each in order to determine synaptic configurations of late meiotic prophase nuclei at the electron microscope (Figure 8). As in *ndj1*Δ, short chromosomes lag in completion of synapsis, as demonstrated by the presence of relatively short single chromosome axes (short arrows in Figure 8), in nuclei composed mainly of synapsed axes (long SCs pointed out by long arrows in Figure 8). We searched for nuclei with the opposite pattern of synapsis in wild-type and in mutant cells, and found none. To do this, we identified spread nuclei with asynapsis either among the longest 3 chromosomes or among the shortest 3 chromosomes but not both. Strikingly, the asynapsed chromosomes were invariably among the shortest 3 chromosomes for wild-type (10 nuclei), *mps3-dAR* (9 nuclei), *mps3-dNT* (6 nuclei), *csm4*Δ (5 nuclei) and *ndj1Δ mps3-dAR* (22 nuclei). This indicates that in budding yeast the shorter chromosomes synapse last, in contrast to larger organisms where the smaller chromosomes tend to synapse first [63]. In addition, single axes frequently are relatively distant from one another, 1 µm or more, suggesting that the failure to synapse is the result of a primary defect in pairing. Thus, in these mutants, pairing and synapsis are delayed without any other obvious abnormalities that might be detected in these preparations, *e. g.*, persistent nonhomologous associations or interlocks.

### Defects in localization of Ndj1 and Csm4 to telomeres in *mps3-dAR* suggest a simple mechanical origin for the RPM defects

**Figure 9:**
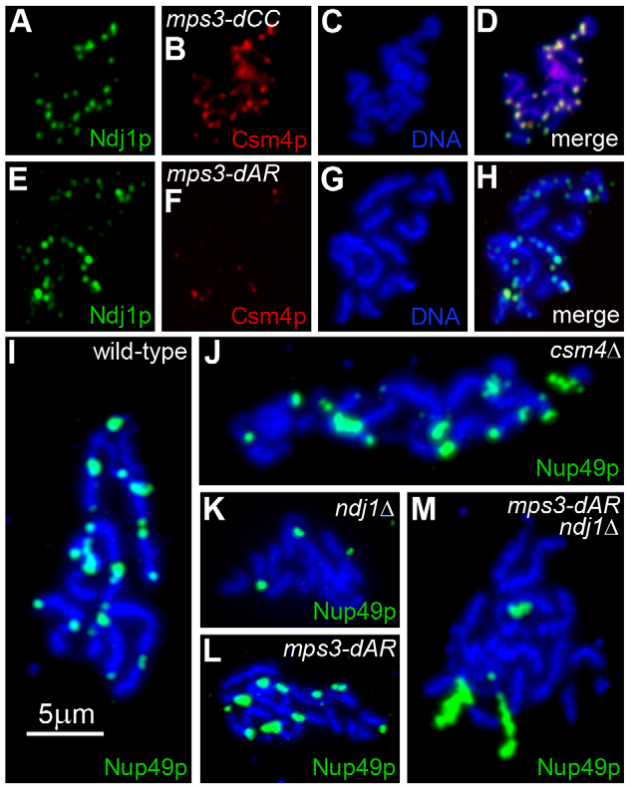
Telomere association with Ndj1 and Csm4, but not with the nuclear envelope, is diminished in *mps3-dAR*. (A–D) Immunolocalization of Ndj1 and Csm4 at telomeres appears wild-type in a spread meiotic prophase nucleus from *mps3-dCC* (MDY2865XMDY2867). (E–H) Immunolocalization in *mps3-dAR* (MDY2868XMDY2870) reveals that colocalization of Ndj1 and Csm4 at telomeres is infrequent and that spots of Ndj1 frequently are found away from the telomeres. (I–M) Nuclear pores, marked by immunolocalization of Nup49-GFP, remain associated with telomeres in spread preparations of nuclei at high frequencies in wild-type (MCY1438XMCY1439) (I) and *csm4*Δ (MDY3449XMDY3450) (J) but not in *ndj1*Δ (MDY2936XMDY2937) (K). Telomere-pore association is frequent in *mps3-dAR* (MDY2952XMDY2953) (L) unless combined with *ndj1*Δ (MDY3445XMDY3447) (M).

The gradation of quantitatively different phenotypes among the mutants suggests a common mechanism with different degrees of impairment. Meiotic defects in *ndj1*Δ, *mps3-dNT* and *csm4*Δ generally have been attributed to defects in the connections between telomeres and motors present in the cytoskeleton. In *ndj1*Δ, proteins Mps3 and Csm4 associate with telomeres sufficiently to promote weak RPMs but in amounts that are difficult to visualize by immunolocalization [9], [27]; in *mps3-dNT*, proteins Ndj1 and Csm4 are undetectable at telomeres and RPMs are absent [9], [27]. In *mps3-dCC*, both Ndj1 and Csm4 accumulate apparently normally at the telomeres (Figure 9A–9D), as expected given the nearly wild-type levels of RPMs in early prophase. The region of Mps3 that is absent in *mps3-dCC* lies between the nuclear membranes and thus shortens the telomere-cytoplasm bridge in the perinuclear lumen, possibly weakening the link to the cytoskeleton. In *mps3-dAR*, accumulations of Ndj1 occasionally are apparent at telomeres though more frequently are found in spots along the chromosome arms, and Csm4 is visualized only in association with the more prominent Ndj1 spots at telomeres (Figure 9E–9H), suggesting that the telomere-cytoplasm link is frequently weakened or absent at telomeres. The significance of the non-telomeric accumulations of Ndj1 is not clear and is being pursued in independent work. Direct immunocytological examination of *mps3-dCC* and *mps3-dAR* proteins has been hampered by severe phenotypes caused by adding epitopes to the mutants (data not shown, and S. Jaspersen, personal communication).

We reported previously that telomere tethering to the nuclear envelope in meiotic prophase in wild-type cells is sufficiently stable during the spreading procedure to maintain telomere association with fragments of nuclear envelope that contain nuclear pores ([9] Supplemental Information Figure 9). We tested wild-type, *ndj1*Δ and *csm4*Δ for telomere-pore association and found the predicted results, a robust association in wild-type and *csm4*Δ (Figure 9I–J) that is lost in *ndj1*Δ (Figure 9K). Telomere-pore association is apparent in *mps3-dAR* (Figure 9L) and requires Ndj1 (Figure 9M), suggesting that telomeres are relatively well anchored. Telomere anchoring is defective in *mps3-dAR* vegetative cells [42], and it seems likely that Ndj1 stabilization of telomere association with *mps3-dAR* protein overcomes this defect in meiotic prophase. Accumulation of Csm4 at telomeres is defective in *mps3-dAR*, for a reason that is not clear, and possibly accounts for the early RPM defects.

### Bouquet formation *per se* is poorly correlated with homolog pairing rates

By standard visual assays, the bouquet stage is essentially absent in the RPM mutants *ndj1*Δ [26], *mps3-dNT* [9] and *csm4*Δ [27]–[29], even though the RPMs and other parameters of meiosis are less defective in *ndj1*Δ than in the other two mutants (as described in reference to Figure 7 and Figure 8, above). Unrelated, pleiotropic effects of the mutants could account for bouquet failure, but it is possible that bouquet formation is particularly sensitive to RPM defects even while other meiosis parameters have a more graded response. To address this question, we analyzed bouquet formation in *mps3-dCC* and *mps3-dAR*.

**Figure 10:**
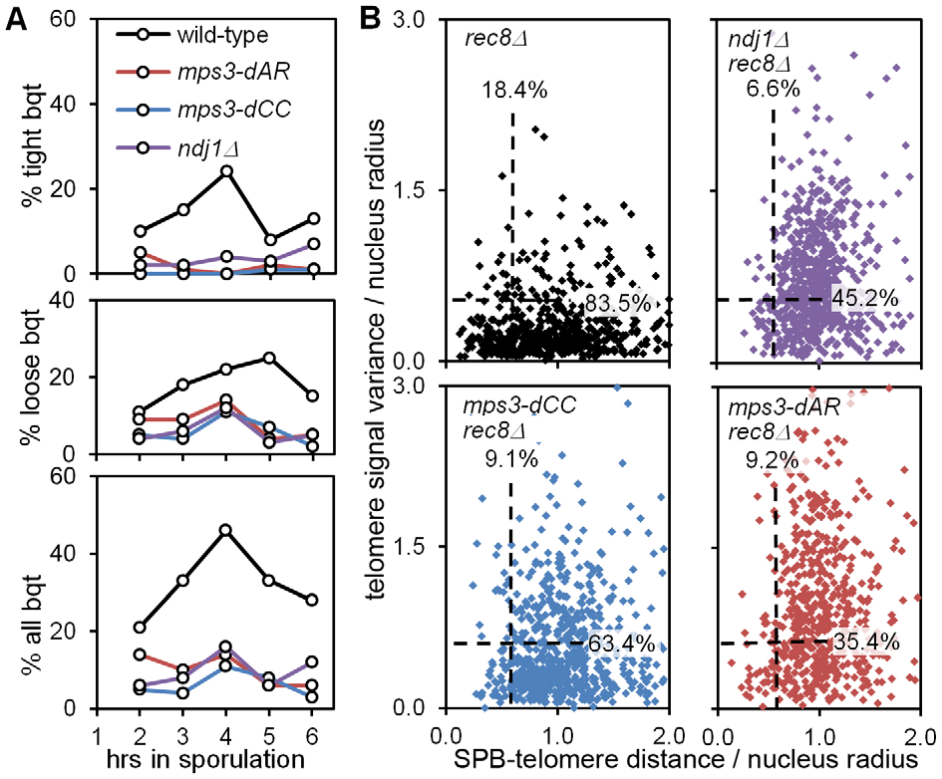
*mps3-dCC* and *mps3-dAR* are defective in bouquet formation and display altered patterns of telomere clustering and distribution relative to the SPB. (A) Bouquet assay strains marked with Spc42-dsRed to visualize the spindle pole body, and with Rap1-CFP to visualize telomeres, are stained with DAPI to visualize the DNA and imaged in 3-dimensional, high-resolution, deconvolved stacks. Results are from single experiments where 200 cells per time-point were scored visually by merging the 3 individual images for each nucleus to generate a 3-color image stack that is then rotated in software to put the spindle pole body at the periphery of the nucleus as viewed in a 2-dimensional projection [9]. Cells are scored as positive when they have a single telomere cluster, within ∼1/5 the apparent nuclear volume of the spindle pole, where the cluster is tight (top panel), loose (middle panel) or either (bottom panel). Strains used: wild-type (MDY2455XMDY2513), *mps3-dAR* (MDY2509XMDY2511), *mps3-dCC* (MDY2558XMDY2560), *ndj1*Δ (MCY1570XMCY1571). (B) Telomere distribution and proximity to the spindle pole are quantified by software in the *rec8*Δ background, where cells blocked in meiotic prophase are found to have a single telomere cluster [48], unless the cells also are mutant for the ability to cluster the telomeres as in *ndj1*Δ and *mps3-dNT* [9]. Each point represents the measurements from a single nucleus, marked and imaged as in (A), above. Dashed lines lie at 0.6 on the respective axes; the associated numbers are the fractions of each population between 0.0 and 0.6. The radius of each nucleus in microns is estimated by the distance in microns from the centroid of the spindle-pole body signal to the centroid of the DAPI signal. Telomere distribution (units of microns) is estimated from the 3-dimensional variance of the Rap1-CFP signal intensity for each nucleus (units of squared microns), normalized by nucleus radius. SPB-telomere proximity (a unitless ratio) is the distance in microns between the centroid of the spindle pole body signal and the centroid of the Rap1-CFP signal, normalized by nucleus radius. Thus, a tight cluster of telomeres adjacent to the spindle pole, as in tight bouquets, would generate a SPB-telomere distance of ∼0, while a tight cluster of all telomeres at the edge of the DAPI signal but opposite the spindle pole would generate a SPB-telomere distance of ∼2. Statistical analysis in the legend to Table 1. Strains used: *rec8*Δ (MDY2517XMDY2534), *ndj1Δ rec8*Δ (MCY1533X1535), *mps3dCC rec8*Δ (MDY2553XMDY2555), *mps3-dAR rec8*Δ (MDY2557XMDY2539).

The standard assay for the bouquet configuration in budding yeast is to visualize the positions of ∼all telomeres with respect to the spindle pole, and to score as having a “tight bouquet” those nuclei with telomeres (1) tightly clustered and (2) in close proximity to the spindle pole. To accommodate the inevitable ambiguity in making this call, nuclei are scored as having a “loose bouquet” when telomeres are on the spindle pole side of the nucleus but not immediately adjacent to the spindle pole and are either loosely or tightly clustered [9], [29]. Tight bouquet nuclei are nearly absent in *mps3-dCC* and *mps3-dAR*, as in *ndj1*Δ, and loose bouquet nuclei are similarly reduced in the three mutants as well (Figure 10A). Thus, by the standard bouquet assay, *mps3-dCC* and *mps3-dAR* are as defective as *ndj1*Δ, the canonical bouquet-less mutant.

We next looked more carefully at telomere behavior in the mutants to question whether the bouquet defect might arise from a defect in clustering or in accumulating at the spindle pole. We have shown previously that deletion of *REC8*, which causes an arrest in meiotic prophase with tightly clustered telomeres [48], provides a sensitized background that reveals an inability to form telomere clusters in *ndj1*Δ and *mps3-dNT* [9]. We quantified telomere distribution and spindle pole to telomere distance in 3D images of nuclei using software routines that automate the measurements. Briefly, image stacks composed of 64 slices at 0.2 micron intervals were deconvolved, smoothed by Gaussian blurring and thresholded to reduce background noise. The distribution of telomeres is estimated as the variance in Rap1-CFP signal per nucleus, the variance being higher when the signal is more wide-spread. The spindle pole to telomere distance is measured by finding the distance in microns between the centroids of the Spc42-dsRed and Rap1-CFP signals. Both these measures are normalized by dividing by the nucleus radius (estimated by the distance between the Spc42-dsRed and DAPI centroids [9]). We found that these measurements generally correspond with visually-identified bouquet nuclei in wild-type samples when both measurements are less than 0.6 for a given nucleus (data not shown). The results are shown with dashed lines at 0.6 for each axis and with the fractions of the populations that fall under each line (Figure 10B; statistical analyses in Table 1).

In agreement with the visual scoring, *mps3-dCC*, *mps3-dAR* and *ndj1*Δ are defective in making “bouquets” in the *rec8*Δ background and, statistically, are indistinguishable from one another (Table 1). However, the phenotypes differ in detail. While telomere proximity to the spindle pole is similarly defective in the mutants, telomere cluster formation in *rec8Δ mps3-dCC* is significantly less defective, and in *rec8Δ mps3-dAR* is significantly more defective, than in *rec8Δ ndj1*Δ. Telomere clustering also is defective in vegetative cells in *mps3-dAR* [42]. The key observation is that chromosome pairing is less defective in *mps3-dAR* than in *ndj1*Δ even though telomere clustering appears more defective in *mps3-dAR*. Thus, telomere clustering and proximity to the spindle pole are, like canonical bouquet formation *per se*, poorly correlated with pairing rates.

## Discussion

Telomere-promoted rapid prophase movements in meiotic prophase first appear in early leptotene as an increase in translation of telomeres across the nuclear envelope, without a concomitant increase in the average speed of movement even though there are occasional, brief movements that are faster than seen in vegetative cells (Figure 1). These early RPMs foster interactions between heterologous as well as between homologous chromosomes, independent of meiotic recombination and prior to zygotene (Figure 3). Two *mps3* mutants with defects in RPMs intermediate between wild-type and *ndj1*Δ also have intermediate chromosome pairing rates (Figure 4, Figure 5, Figure 6) and in general have intermediate rates of sporulation and spore viability, disomic spore production, premature sister chromatid separation and, for *mps3-dAR*, delay prior to anaphase I (Figure 7). Among RPM mutants with a range of delays in completing synapsis, as in wild-type, we consistently observe that shorter chromosomes are the last to synapse (Figure 8). Given that (1) recombination generally is not reduced in RPM mutants, and (2) the normal mechanisms that lead to synapsis are likely intact [28], [29], [64], [65], we conclude that the delay in synapsis results primarily from a delay in pairing, consistent with prior work similarly supporting a role in pairing for Ndj1 [66] and Csm4 [30].

Immunocytological examination suggests that the common defect among the various RPM mutants is that telomeres do not engage cytoplasmic motors as in wild-type cells, either because attachments to the SUN-protein bridge across the nuclear envelope are weakened (*ndj1*Δ, *mps3-dAR*, *mps3-dNT*; Figure 9), the bridge itself is defective (*mps3-dCC*) or the motors associated with the cytoskeleton are somehow rendered ineffective (*csm4*Δ). Surprisingly, as assayed here formation of the bouquet appears to be equally defective in the various RPM mutants (Figure 10). It is particularly informative that pairing in *mps3-dCC* appears only slightly delayed, (Table 1), suggesting that the bouquet makes at most a small contribution to pairing in budding yeast. Rather, the kinetics of pairing are strongly correlated with the ability of the telomere-led movements to change the locations of the chromosomes within the nucleus. We suggest that telomere translation along the nuclear envelope is the critical feature of the SUN protein-promoted movements, in part because deformations of the yeast nucleus reported by others [5], [28], [31] are relatively mild and infrequent in our strains. In *mps3-dNT* and *csm4*Δ, where movements are equivalent to the level in mitotic cells [27], pairing and synapsis presumably are aided by movement from activities such as thermal motion, chromatin remodeling, DNA and RNA metabolism, and polymerization/depolymerization of intranuclear microtubules that might displace chromatin.

We anticipated a pairing function for RPMs [27] and, with others, suggested that RPMs may in addition promote destabilization of inappropriate interactions, entanglements and/or interlocks [12], [27], [29], [31]. We have not observed interlocked chromosomes in our spread preparations. Furthermore, longer chromosomes would seem more susceptible than short chromosomes to entanglements and interlocks, and a defect in resolving these problems could be expected to delay long chromosome pairing and/or synapsis disproportionately. However we have observed that smaller chromosomes are the last chromosomes to pair and synapse in the RPM mutants (or perhaps never do synapse, as chromosomes that lack crossovers in *ndj1*Δ are the shorter ones - see Table S7 in [65]). The simplest interpretation is that RPMs primarily influence pairing and synapsis not by resolving interlocks but by extending the range of the homology search. Nevertheless, interlocks may be difficult to visualize in budding yeast and, furthermore, we cannot rule out the possibility that RPMs contribute to interlock formation by promoting telomere-proximal recombination at an early stage and then contribute to interlock resolution by continued movement at later stages. It also remains possible that RPMs disengage less cytologically evident entanglements, such as entanglements between chromosome axes which are resolved prior to the onset of synapsis or entanglements of loops of chromatin of different chromosomes, which would not involve the chromosome axes [27].

Conservation of the bouquet suggests conserved function and, given the timing of bouquet formation it is not surprising that a role for the bouquet in pairing is widely accepted. A complicating factor for earlier work is that prior to the recognition that telomere-led RPMs are well-conserved, bouquet formation appeared to be the primary defect in a variety of mutants with pairing and synapsis defects, a good example being *ndj1*Δ where it now appears that the RPM defect is primary, with bouquet and pairing defects being secondary. Fission yeast, which has provided the clearest data in support of a role for the bouquet in pairing and recombination, also has provided the clearest data for a role for the bouquet in SPB stability and spindle function in the first meiotic division [67]. The sporulation and spore viability defects in *mps3-dCC* could reflect a direct impact of the mutation on spindle pole body function *per se*, given that the defects in RPMs and chromosome segregation are mild or absent (Table 1). However, we have observed no defects in vegetative growth in *mps3-dCC*, and it is possible that absence of the bouquet in *mps3-dCC* specifically causes the later problems.

**Figure 11:**
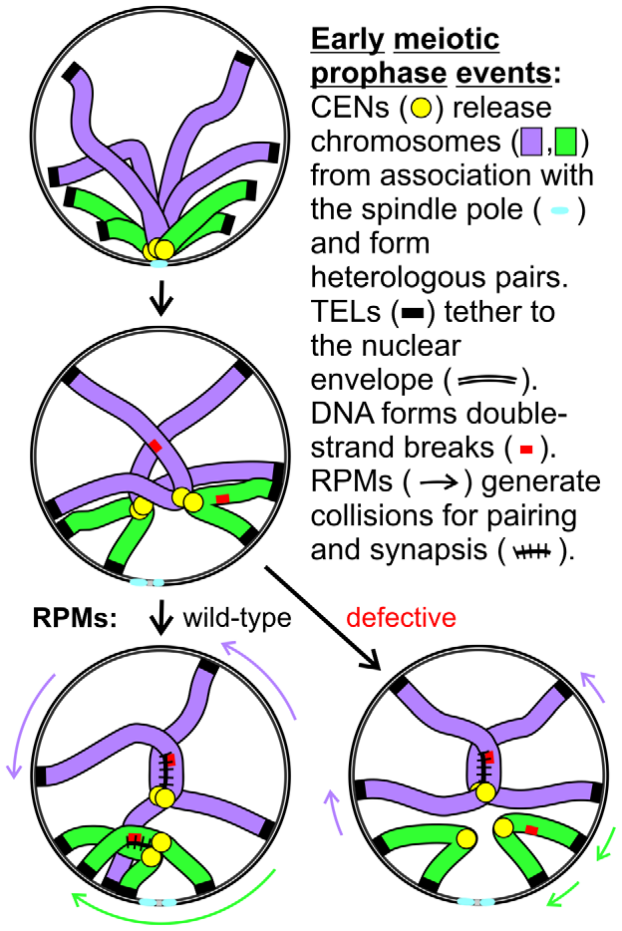
Model for RPM contribution to pairing. Two pairs of homologs are diagrammed but only one chromatid is shown for each chromosome. Centromeres remain in close proximity following dissolution of the Rabl orientation (the first change diagrammed) due to a centromere-specific mechanism that joins pairs of nonhomologous centromeres but telomere anchoring to the nuclear envelope can hold chromosome arms apart. Short chromosomes depend more than long chromosomes on long range telomere movements that allow and generate collisions that in turn promote homology assessment, stabilization of association and synapsis.

Specifically how RPMs function to promote pairing in combination with the recombination-directed homology search is an open question. Following the DNA double-strand break (DSB) formation that launches meiotic recombination, resection creates single-stranded DNA that is coated with *recA*-like enzymes which then promote invasion of homologous DNA, this last step presumably insuring that pairing is homology-dependent. The farther the single-strand end can diffuse away from the axis of the chromosome, the less dependent on active whole-chromosome movement this part of the search would become, although presumably the potential for entanglements would increase as well. Reliance of timely pairing and synapsis on RPMs suggests that the single-strand extension search volume is limiting. A simple model for the role of RPMs early in prophase is that RPMs promote collisions by generating relatively random long-range chromosome movements, thus increasing the probability that a single-stranded end will encounter homologous DNA [45], [68], [69]; this process might be particularly important for short chromosomes that cannot reach across the nucleus when telomeres are tethered to the nuclear envelope (Figure 11).

The meiotic delay associated with defective RPMs leads to negative consequences for the cell, although the mechanism is not certain. One possibility is that the pairing delay leads to continued resection which might help promote the homology search by extending the search radius but at a cost of increased entangling and/or possibly of increased ectopic recombination [54]. Alternatively, checkpoint adaptation before recombination is sufficiently complete could lead to chromosome missegregation, or depletion of energy stores during the prolonged prophase could prevent completion of sporulation. Whether RPMs play additional roles in meiotic chromosome metabolism remains to be determined but their conservation across phyla indicates that RPMs are critical for normal meiotic outcomes and fertility.

## Materials and Methods

### Strains and plasmids

Strains and plasmids are described in Table S1. For strains marked with GFP spots, pMDE798 and pAFS152 (provided by A. Straight and A.W. Murray) were transformed into MDY strains to generate *DMC1* and *CYC1* promoter-driven lacI-GFP expression respectively. A tandem array of 256 copies of the lac operator (LacO_256_) was then targeted at the desired locus (at chromosome *1*L, *5*L or *7*L mid-arm; *1*, *5* or *7* centromere, *1*R, *3*L, *4*R, *5*L or *7*L telomere) into strains containing P_DMC1_- and/or P_CYC1_-lacI-GFP [9], [27], [70]. Standard genetic procedures were applied to generate various single and double mutants for movement analyses, and for bouquet and pairing assays, mainly by crossing appropriate single mutants followed by dissecting tetrads to identify further haploids in isogenic backgrounds. Isogenic haploid clones of opposing mating types were mated, and zygotes were selected on appropriate medium (adenine-) to get homozygous diploid clones which were then synchronized for sporulation. Partial deletion alleles of *MPS3*, *mps3-dAR* (residues 65–145 deleted) and *mps3-dCC* (residues 240–430 deleted) were constructed by PCR and sequenced before use. The mutant alleles were cloned into the *URA3* vector YIplac211 and integrated at the genomic site of *MPS3*. Successful replacements of *MPS3* with the deletion alleles were identified by PCR screening of 5-FOA^R^ colonies.

### Fluorescence microscopy

Fluorescence microscopy of living cells was carried out as described previously [9], [27], [46]. Briefly, an agarose pad was used to trap sporulating cells against the bottom of the coverslip for time-lapse microscopy [71] using a rapid, thru-focus method that produces images projected onto a single plane for each acquisition [46]. For bouquet analyses, cells were briefly fixed with 0.4% paraformaldehyde, mounted as for live cell microscopy, and imaged in high-resolution, deconvolved 3D stacks [9]. Fluorescent spot movements and distributions were analyzed using algorithms and software developed for the purpose. The efficiency of spot movements was estimated by measuring projected area over 20, 60 or 120 second intervals [27]. Spread meiotic nuclei for immunofluorescence were prepared on poly-L-lysine coated slides [72].

### Assays

Sporulations for all cytological assays were carried out in liquid medium using standard procedures [70]. Strains containing tetramerizing-lacI-GFP were kept in 20 mM IPTG through all stages of handling until shifting into sporulation, in order to prevent rearrangements and losses of the LacO concatemers. Bouquet assay strains labelled with Rap1-CFP to mark telomeres and Spc42-dsRED to mark the spindle pole body were fixed briefly with 50% ethanol containing DAPI to label DNA [9].

### Statistical analysis

The significance of differences between non-normally distributed datasets was assessed using the Kolmogorov-Smirnov test, calculated at http://www.physics.csbsju.edu/stats/, or the Mann-Whitney U test in Excel (Microsoft), downloaded from http://udel.edu/~mcdonald/statkruskalwallis.html. Chi-square tests using Yates' continuity correction were calculated at http://udel.edu/~mcdonald/statchigof.html and Student's t-tests (2-tailed, 2-sample unequal variance) were calculated using Excel.

## Supporting Information

Figure S1 Pairing rates are positively correlated with RPMs. Pairing rates (Figure 4, Table 1) and RPM measures (Figure 5, Table 1) are graphed to display the relationship of pairing behavior of individual genotypes with respect to each of the 4 RPM parameters for unpaired (A–D) and paired (E–H) telomeres *4*R. Paired telomeres tend to move faster and further than unpaired telomeres in wild-type, *mps3-dCC* and *mps3-dAR* but the reverse is true for average and maximum speeds in *ndj1*Δ (see [27] and Figure 5). Error bars in all graphs are average absolute deviation from the median; horizontal bars are for the RPM measures and vertical bars are for the pairing rates by genotype (see Figure 6B). Strains used are listed in Table S3.
(TIF)

Table S1 Strains and plasmids.
(XLS)

Table S2 Strains used in Figure 3.
(XLS)

Table S3 Strains used in Figure 4.
(XLS)

Video S1 Time-lapse images of wild-type, *mps3-dCC*, *mps3-dAR* and *ndj1*Δ at 4 hours. Shown are fields containing 20–25 cells/nuclei with each of the 4 genotypes, where the real time between movie frames is 1 second. Each nucleus contains a larger, fuzzier spot that marks the spindle pole-associated microtubules and 1 or 2 smaller, discrete spots that mark a pair of homologous telomeres (1 spot following pairing/synapsis that causes fusion of the 2 spots). Telomere movements tend to be more vigorous following pairing/synapsis, *i. e.*, in the 1-telomere-spot *versus* the 2-telomere-spots nuclei, and in wild-type cells as compared with *mps3-dCC*, *mps3-dAR* and, most noticeably, *ndj1*Δ. Quantification generally is required to make clear distinctions between different movement types, mutants and time-points. Strains used: wild-type (MDY1560XMDY1567), *mps3-dCC* (MDY2580XMDY2759), *mps3-dAR* (MDY2523XMDY2756), *ndj1*Δ (MDY2294XMDY1560). Method: Concatemers containing 256 copies of the lacI binding site from LacO were inserted adjacent to the telomeres of both chromosome *4* right arms and were decorated with lacI-GFP to produce discrete fluorescent spots marking the positions of the telomeres in living cells. Microtubules were labeled with Tub1-GFP which marks the position of the spindle pole as a fuzzy spot that generally is larger than the telomere-adjacent spots. Images were acquired every second for 1 minute using a Zeiss Axioplan 2ie fitted with a 100× 1.4NA objective, Roper CoolSnap camera and custom-written software to acquire “thru-focus” images which were deconvolved using a constrained iterative algorithm [46]. Movies were analyzed using custom-written software [27].
(MOV)
