# Supplementary figures and images for: Meiotic Chromosome Pairing Is Promoted by Telomere-Led Chromosome Movements Independent of Bouquet Formation
Source: PLoS Genet. 2012 May 24;8(5):e1002730. doi: 10.1371/journal.pgen.1002730 (PMC3359977; doi:10.1371/journal.pgen.1002730)

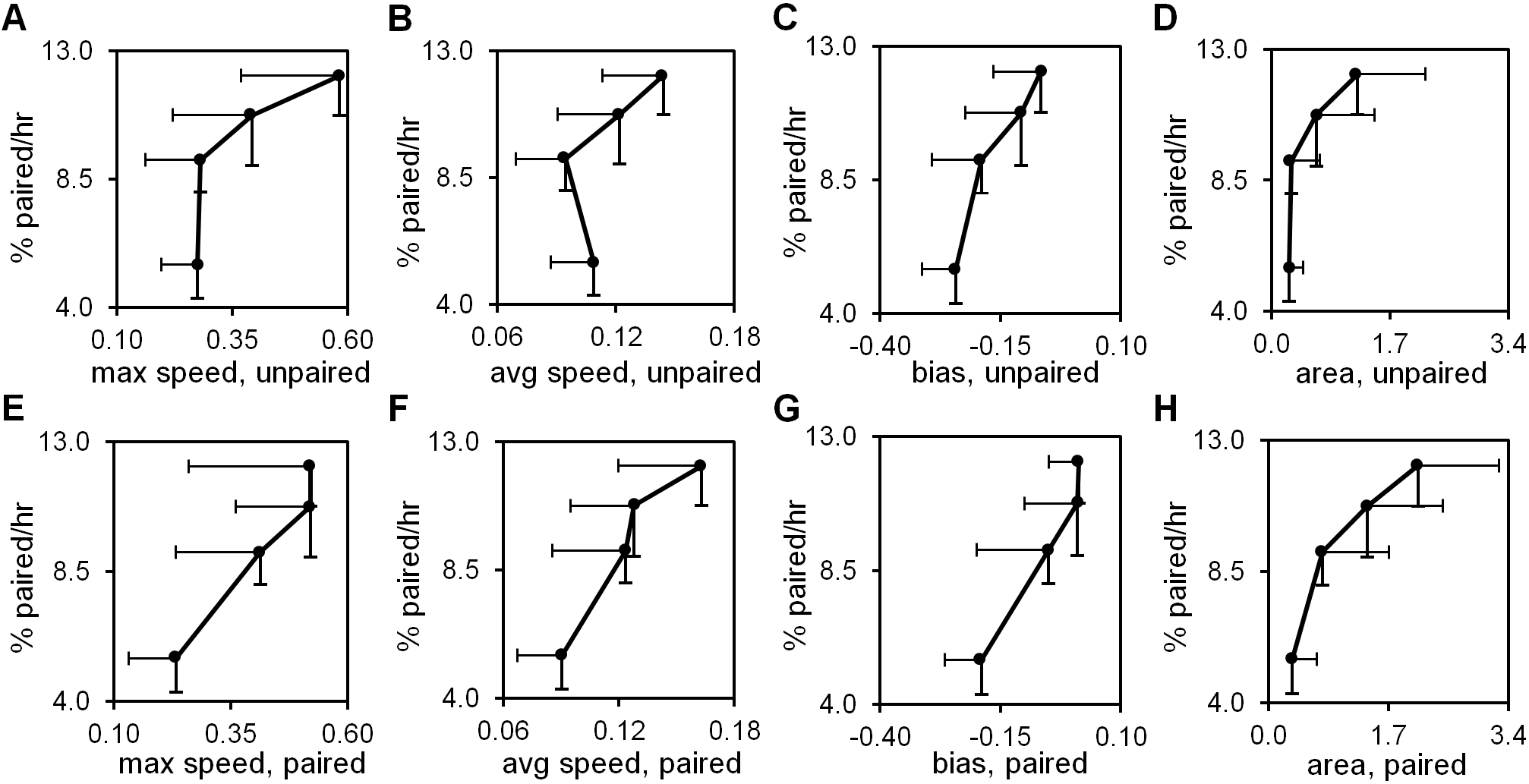

Supplement: Figure S1 — Pairing rates are positively correlated with RPMs. Pairing rates (Figure 4, Table 1) and RPM measures (Figure 5, Table 1) are graphed to display the relationship of pairing behavior of individual genotypes with respect to each of the 4 RPM parameters for unpaired (A–D) and paired (E–H) telomeres 4R. Paired telomeres tend to move faster and further than unpaired telomeres in wild-type, mps3-dCC and mps3-dAR but the reverse is true for average and maximum speeds in ndj1Δ (see [27] and Figure 5). Error bars in all graphs are average absolute deviation from the median; horizontal bars are for the RPM measures and vertical bars are for the pairing rates by genotype (see Figure 6B). Strains used are listed in Table S3. (TIF) [file pgen.1002730.s001.tif]
